# Accuracy of Full-Guided and Half-Guided Surgical Templates in Anterior Immediate and Delayed Implantation: A Retrospective Study

**DOI:** 10.3390/ma14010026

**Published:** 2020-12-23

**Authors:** Yuan Chen, Xiaoqing Zhang, Maoxia Wang, Qingling Jiang, Anchun Mo

**Affiliations:** 1 State Key Laboratory of Oral Diseases, Department of Implant Dentistry, West China Hospital of Stomatology, Sichuan University, Chengdu 610041, China; chenyuanwestchina@stu.scu.edu.cn (Y.C.); 2017224035151@stu.scu.edu.cn (X.Z.); 2018324035051@stu.scu.edu.cn (M.W.); 2 Department of Epidemiology and Medical Statistics, West China School of Public Health and West China Fourth Hospital, Sichuan University, Chengdu 610041, China; jiangql@stu.scu.edu.cn

**Keywords:** full-guided, half-guided, immediate implant placement, delayed implant placement, accuracy, anterior, computer-aided implantology

## Abstract

Computer-aided implantology has developed rapidly in recent years, this study aimed to compare the accuracy of guided-surgery between anterior immediate and delayed implantation, and simultaneously assess the effect of full-guided and half-guided templates on accuracy values. Seventy-six implants were inserted in 63 patients using full-guided or half-guided template in the anterior zone. Postoperative cone beam computed tomography (CBCT) was matched with preoperative planning to evaluate the deviation between actual and planned implants. No statistical difference was found in any deviation between immediate and delayed implantation (*p* > 0.05). In anterior immediate implantation, the global coronal, apical, depth and angular deviations of full-guided templates were all significantly lower than those of half-guided templates (0.66 ± 0.26 vs. 1.10 ± 0.76 mm, 0.96 ± 0.41 vs. 1.43 ± 0.70 mm, 0.46 ± 0.24 mm vs. 0.93 ± 0.79 mm and 1.69° ± 0.94° vs. 2.57° ± 1.57°). While in delayed implantation, full-guided templates only perform better with statistical significance on global apical and depth deviation (1.01 ± 0.42 vs. 1.51 ± 0.55 mm and 0.32 ± 0.26 vs. 0.71 ± 0.47 mm). After excluding the influence of depth deviation, the coronal and apical deviations between the two systems in immediate implantation and the apical deviations in delayed implantation had no statistical difference. Within the limit of this study, the results suggested the accuracy of guided-surgeries for anterior immediate and delayed implantations was comparable, and full-guided template was more accurate for immediate and delayed implantation.

## 1. Introduction

Over the last decades, the computer-aided implantology (CAI) has drawn more and more attention with the potential for a more accurate, predictable and less invasive surgery [1,2,3]. Furthermore, unlike traditional free hand surgeries based on the operators’ experience and imagination, CAI followed the principle of “prosthesis-driven” implant placement, creating an ideal situation for anterior aesthetic restorations [4,5]. Commercially planning software such as SimPlant^®^ and Nobel Clinician^®^, in combination with three-dimensional (3D) tissue information obtained from cone beam computed tomography (CBCT) and optical scan, opened the possibility for adequate preoperative design [6,7,8,9,10]. However, the data acquisition and virtual planning were only the preoperative stages of CAI, and it was the essence to find a reliable transfer approach to guide the surgical procedure [11]. Recently, due to the rapid development of computer-aided design/manufacturing (CAD/CAM) and 3D printing techniques, the presurgical planning could be transferred to the clinical situation by static surgical templates, allowing the clinicians to reproduce the virtual treatments intraoperatively [12,13].

Although the software and hardware have been much improved since the application of surgical template in the early 1990s [14], the accuracy of guided-surgery was still controversial and deviation may occur in every step within the workflow of CAI [15,16,17,18,19,20,21,22]. According to whether a guided cylinder was needed in the final implant installation, the surgical templates could principally be divided into half-guided and full-guided systems [23]. Inconsistent conclusions were drawn in different researches on the accuracy of the two different templates, and they were mainly limited to edentulous and posterior areas [19,24,25,26]. The comparison of accuracy between the two templates in anterior area has not been done so far. The main purpose of this study was to compare the accuracy of guided-surgery between anterior immediate and delayed implantation, and simultaneously evaluate the influence of half-guided and full-guided template on accuracy values, aiming to provide clinicians a better choice in real operative procedures.

## 2. Materials and Methods

### 2.1. Patient Selection

Sixty-three patients who received computer-guided surgery of anterior teeth between June 2017 and July 2019 in West China Hospital of Stomatology Sichuan University, China, were included in this study. Patient selection was conducted on the basis of the following inclusion and exclusion criteria.

Inclusion criteria:

1. 18 years of age or older;
2. At least 1 tooth having missed within 3–4 months or to be extracted in anterior zone, and the remaining teeth are adequate to support the surgical template;
3. Sufficient bone tissue to insert an implant without any need for augmentation (the residual bone height ≥ 10 mm, the buccolingual width ≥ 7 mm, and the labial bone plate is complete without bone dehiscence and fenestration);
4. Good treatment compliance.

Exclusion criteria:

1. Presence of uncontrolled systemic diseases, infection or inflammation around the implant sites;
2. Pregnancy or lactation;
3. Poor oral hygiene habits;
4. Psychiatric problems, alcohol, tobacco (>10 cigarettes per day) or drug abuse;
5. Severe bruxism or clenching;
6. Not able to complete the follow-up.

All subjects gave their informed consent for inclusion before they participated in the study. The study was conducted in accordance with the Helsinki Declaration of 1975 as revised in 2013 and approved by the local ethical committee (WCHSIRB-ST-2017-113).

### 2.2. Data Acquisition

All included patients got a CBCT scan (3D Accuitomos^®^, Morita, Kyoto, Japan) with the setting as follows: 90 kVp, 5 mA, 17.5 s, voxel size 0.25 m, slice thickness 0.25 mm and field of view (FOV) 140 mm × 100 mm, and these data were converted into Digital Imaging and Communications in Medicine (DICOM) format for bone reconstruction. Then, patients received oral scan impression (TRIOS 3^®^, 3 Shape, Copenhagen, Denmark) to get intraoral information including soft tissue and teeth. The virtual prosthesis design was conducted on the digital software (3 Shape Dental System^®^, 3 Shape, Copenhagen, Denmark), which was finally converted to surface tessellation language (STL) format files.

### 2.3. Implant Planning Procedure and Template Fabrication

**Figure 1:**
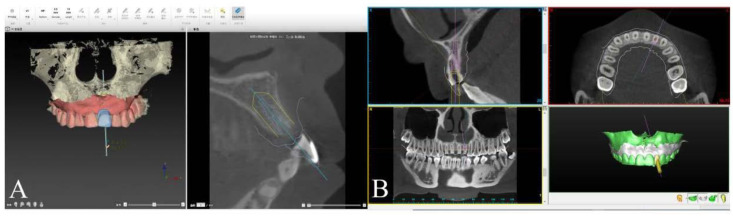
Virtual implant placement and treatment planning in digital software. (**A**) Full-guided surgical template was designed in Nobel Clinician^®^ and (**B**) half-guided surgical template was designed in SimPlant^®^.

**Figure 2:**
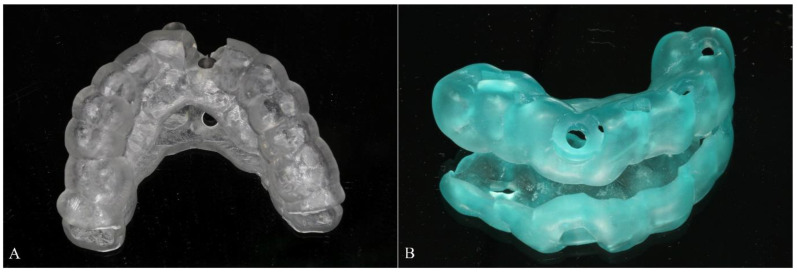
All surgical templates were tooth-supported. (**A**) Full-guided template and (**B**) half-guided template.

The CBCT data in DICOM format was imported to implant planning software (full-guided template: Nobel Clinician^®^, Nobel Biocare AB, Gothenburg, Sweden; half-guided template: SimPlant^®^, Materialise, Leuven, Belgium) for jaw reconstruction. The intraoral scanning data and prosthesis design in STL files were also imported and matched the CBCT data by aligning their mutual teeth. After finishing the 3D reconstruction, virtual implant planning was performed following the ITI treatment guide (Figure 1A,B). Thereupon, both full-guided and half-guided template data were calculated and exported as STL file to the corresponding factory for 3D printing (ProJet MJP 2500/2500Plus, MIRADUR, Guangzhou, China), which could produce the designed model through sequentially printing 2D layers of resin on top of one another. All templates used in our research were tooth-supported (Figure 2A,B).

### 2.4. Surgical Procedure

**Figure 3:**
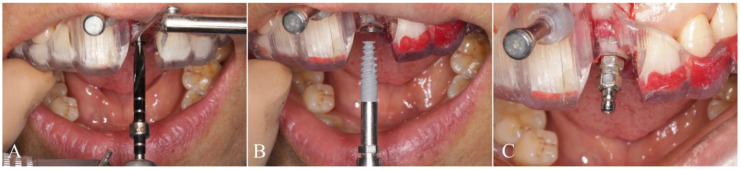
Surgical procedure with full-guided surgical template. (**A**) Implant cavity was prepared with the drill and drill handle under the guide of template; (**B**) implant was inserted with the implant driver under the guide of template and (**C**) depth was controlled by the implant driver.

**Figure 4:**
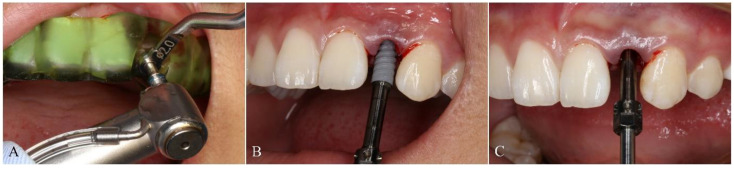
Surgical procedure with half-guided surgical template. (**A**) The implant preparation with the drill under the guide of template; (**B**) implant was inserted with free hand and (**C**) depth was controlled by visual estimation.

The included patients received oral hygiene preoperatively and the seating of templates was verified before surgery was initiated to avoid the positioning errors. All surgeries were conducted by the same experienced operator. Local anesthesia using articaine with adrenaline 1:100,000 was administrated in surgical areas. When the anesthesia has taken effect, operator extracted the tooth scheduled to be removed for immediate implant placement or made a flap directly at the implant site for delayed implantation. The full-guided surgery from the initial drill to the final insertion was conducted using NobelActive^®^ Guide Drilling Kit (Nobel Biocare AB, Gothenburg, Sweden) with a surgical template. The drill handle and implant driver from the kit could control the depth of the drill and implant respectively (Figure 3A–C). In the half-guided surgery, the preparation for implant cavity was conducted by Segma^®^ Guide Drilling Kit (Segma, Beijing, China) using a template, while the final implant insertion was performed using NobelActive^®^ Surgery Kit (Nobel Biocare AB, Gothenburg, Sweden) with free hand (Figure 4A–C). No instrument was used to control the implant depth, which had to be evaluated visually. All implants inserted here were NobelActive^®^ (Nobel Biocare AB, Gothenburg, Sweden) with various dimensions. Every implant in this study showed an excellent primary stability of at least 35 Ncm and immediate restoration was made accordingly.

### 2.5. Accuracy Measurements

**Figure 5:**
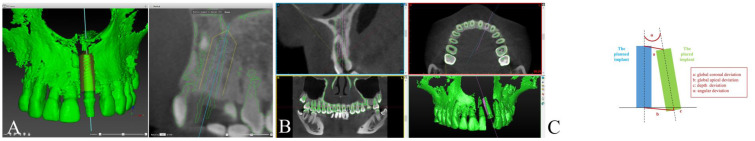
The match between the planned and placed implants. (**A**) The image match in Nobel Clinician^®^; (**B**) the image match in SimPlant^®^ and (**C**) the measurement of global coronal, apical, depth and angular deviation between placed and planned implants. a = global coronal deviation, the liner distance between the planned and placed implants at the coronal; b = global apical deviation, the liner distance between the planned and placed implants at the apical; c = depth deviation, the vertical distance between the planned and placed implants at the apical; α = angular deviation, the angle between the planned and placed implant axes.

Postoperative CBCT was taken immediately after the surgery using the same parameters as preoperative scans and imported in the digital software (Mimics^®^, Materialise, Leuven, Belgium) for 3D reconstruction. Subsequently, the postoperative model was superimposed on the preoperative CBCT image in the corresponding design software (SimPlant^®^ or Nobel Clinician^®^), which contained the information of planned implant (Figure 5A,B). The pre- and postoperative data were matched using automated tools at first, and then they were aligned by superposing some anatomic landmarks (range 5–8) manually. The plane with the largest overlap area between planned and actual implant was selected as the measurement plane, and the following parameters were evaluated: global coronal/apical deviation determined as the liner distance between the coronal/apical center of the planned and actual implant positions, depth deviation measuring the vertical distance between the apical centers of the planned and actual implants, and angular deviation, which was calculated as the angle between the two respective long axis (Figure 5C). A positive-valued deviation in depth meant the actual implant position was higher than the planned sites, while a negative value indicated the implant was inserted too deep. Actually, the global coronal and apical deviation could be decomposed into the deviations parallel to the long axis of the implant and the deviations in the horizontal plane. In order to further analyze the horizontal coronal and apical deviation, we introduced a statistic method to exclude the influence of implant depth on the final results of the liner coronal and apical deviations.

### 2.6. Statistical Analysis

Data analysis was performed using statistical analysis system R-3.5.1. Continuous variables were described using mean, minima and maxima and standard deviation (SD), while categorical variables were showed by frequency and percentage. Normality test results indicated that all the concerned continuous variables could be regarded as normally distributed, except for global coronal deviation, thus the comparison of global coronal deviation between groups was carried out by a Wilcoxon rank sum test, while an independent *t*-test was used for other continuous variable comparison and χ^2^ test for categorical variable comparison. Ordinary linear regression model was used to eliminate the effect of depth deviation on global coronal and apical deviation, to compare the horizontal coronal and apical deviation between groups. *p* < 0.05 was considered statistically significant.

## 3. Results

### 3.1. Details of Included Patients

As shown in Table 1, a total of seventy-six implants were placed in sixty-three patients without any complication, and thirteen patients received two implants at a time. Of these, thirty patients used full-guided surgical templates and thirty-three used half-guided ones. Additionally, thirty-two patients were included in the immediate implantation group and thirty-one received delayed implant placements. More details of the sex ratio, age and implant sites in different groups were visible in Table 1.

Taking implant as a unit for statistical analysis, no significant difference was found in age and gender between any of the following groups (full-guided vs. half-guided; immediate vs. delayed; full-guided in immediate vs. half-guided in immediate; full-guided in delayed vs. half-guided in the delayed group).

### 3.2. Accuracy Measurements

As shown in Table 2, the mean global coronal, global apical and depth deviation of immediate implantation were 0.91 ± 0.63 mm, 1.23 ± 0.63 mm and 0.73 ± 0.66 mm respectively, which were slightly higher than those of delayed implantation group at 0.71 ± 0.41 mm, 1.23 ± 0.54 mm and 0.49 ± 0.41 mm. While the mean angular deviation of immediate implantation was lower than that of delayed implantation. No statistical difference (*p* > 0.05) in any deviation type could be found between anterior immediate and delayed implantation groups.

When comparing the deviation of different surgical templates irrespective of implant timing, it was obvious the implant position achieved using the full-guided template was more accurate than that using the half-guided template, and all these data were statistically significant (Table 2). Specifically, the mean deviation of global coronal, global apical, depth and angular for full-guided surgical templates were 0.59 ± 0.28 mm, 0.99 ± 0.41 mm, 0.38 ± 0.26 mm and 1.91° ± 1.02° respectively, which were all lower than 1.04 ± 0.64 mm, 1.46 ± 0.64 mm, 0.84 ± 0.68 mm and 2.77° ± 1.72° for half-guided templates. Regarding the immediate implant placement, the mean global coronal, global apical, depth and angular deviations of full-guided surgical templates were 0.66 ± 0.26 mm, 0.96 ± 0.41 mm, 0.46 ± 0.24 mm and 1.69° ± 0.94°, which were also statistically significantly lower than those of half-guided surgical templates at 1.10 ± 0.76 mm, 1.43 ± 0.70 mm, 0.93 ± 0.79 mm and 2.57° ± 1.57° respectively. In delayed implant placement, full-guided surgical templates demonstrated a better accuracy with a statistical difference when the global apical and depth deviations were considered. However, the difference between the two modalities concerning the mean global coronal and angular deviations was not significant, with 0.53 ± 0.29 mm and 2.09° ± 1.07° for full-guided templates and 0.94 ± 0.43 mm and 3.06° ± 1.92° for half-guided templates.

Furthermore, to avoid the influence of implant depth on the liner distance between the planned and placed implants at the coronal and apical, we used ordinary linear regression, adjusting the results for depth errors. As shown in Table 3, when comparing full-guided with half-guided templates, which had the same depth deviation, there was an expected difference of −0.012 mm, −0.126 mm and −0.052 mm in coronal deviation, and −0.143 mm, −0.345 mm and −0.206 mm in apical deviation for immediate implantation, delayed implantation and total case, respectively. However, all these differences were not statistically significant (*p* > 0.05), implying the horizontal coronal/apical deviations of full-guided and half-guided templates were similar after excluding the influence of depth deviation.

## 4. Discussion

With the guidance of template, CAI could realize prosthetically ideal implant insertion in the anterior esthetic zone. Besides, the benefits of guided-surgery such as high efficiency, low pain and less bone loss made it more and more popular in clinical practice [3]. However, some research have indicated the accuracy of guided-surgery was still controversial, relevant to supporting approaches (bone, teeth and mucosa), template type (full-guided and half-guided) and operator’s experience [15,16,17]. Different from half-guided templates only directing the drilling bone sequence, the full-guided ones were used in the whole surgical procedure including the final implant insertion. Conventionally, full-guided surgery was more expensive and limited by interocclusal distance [25]. Furthermore, some implant systems did not allow for full-guided insertion. Although some authors reported more accuracy with full-guided templates, the others claimed the accuracy of full-guided and half-guided was comparable [19,23,26]. Until now, the accuracy of the two different templates in the anterior esthetic zone has not been compared, even though it was important for the clinical decision. The purpose of this study was to investigate the accuracy of guided-surgery between anterior immediate (implant was placed immediately after tooth extraction) and delayed implantation (implant was placed within 3–4 months after tooth extraction), and assess the effect of full-guided and half-guided templates on accuracy values in different implanting timings, to determine which was the best choice in clinical practice.

In this study, the immediate and delayed implantation in anterior esthetic zone shown similar accuracy irrespective of template types. Although the delayed implant placement shows better results on linear deviations compared with immediate implantation, it had a worse control of angular deviation. The result of mean deviation at the apex of implants was higher than that at the coronal, and a possible explanation may be the fact that the angular errors increased with the drilling farther into the bone, contributing to a larger value for apical deviation. For the influence of template type on linear and angular deviations, full-guided templates show better accuracy in the anterior esthetic zone with statistically lower value in all deviation parameters. After adjusting global coronal and apical deviation with depth error, the results indicated full-guided surgeries only performed better in the depth and angle for immediate implantation and achieved less depth errors in delayed implantation. According to these results, it could be concluded that the difference of full-guided and half-guided surgical template on global coronal and apical deviation in anterior immediate implantation and global apical deviation in anterior delayed implantation was mainly caused by depth errors. Additionally, the major inaccuracy of half-guided surgeries occurred with the implants not being placed deep enough and its angular errors, which may induce the prosthetic problems especially in anterior esthetic area. Considering the cost-effectiveness and large interocclusal distance required for full-guided templates, the half-guided surgery may be a good choice in some anterior implant cases when the implantation depth and angle were well-controlled. Apart from the mean values, the data of maximum deviation also had directive significance in clinical practice, indicating the minimum linear safety distance that have to be maintained to avoid harming adjacent structures. For example, it would be prudent to leave at least 2.93° for full-guided and 4.49° for half-guided surgeries respectively, based on the angular deviation obtained in this study.

To date, many research about the accuracy of guided-surgeries in edentulous and posterior areas have been published. Consistent with our results, a retrospective study conducted by Cassetta et al. indicated better accuracy was provided by full-guided templates when coronal and depth deviation were considered [1]. It has also been reported in the 4th EAO consensus conference that full-guided implantation had higher accuracy [15]. While Kuhl et al. concluded that there was no significant difference on the accuracy between full-guided and half-guided implantation based on a cadaver study [25]. One of the reasons for the inconsistent results among these studies might be the difference in research models. Commonly, in-vitro and cadaver studies had optimized conditions for implantation, while in clinical cases, doctors often faced more complicated situations that may influence the final accuracy, such as bleeding and saliva [27]. On the other hand, different measuring methods would also influence the outcomes. For example, some researchers measured the liner distance of coronal/apical center between the planned and placed implant for global coronal/apical deviations as we did. While some researchers measured the horizontal distance at the level of the implant shoulder and apex. Currently, there was no standardized index used to measure the accuracy of implantation and different methods were not equal, thus complicating the comparison among different researches.

As mentioned in previous parts, guided implant surgery involved a sequence of preoperative procedures such as data acquisition, treatment planning and the fabrication of templates via CAD/CAM or 3D printing [11,13]. The positional errors for actual implants may occur because of the accumulating errors within the digital workflow [13,27]. Research had proved that the deviations of implantation were mainly related with the preoperative phase rather than surgical procedure [28]. For example, although it has been reported that CBCT was reliable in liner measurements, some factors such as metallic restorations and patient’s movements could induce the image distortion and quality degradation, causing inaccurate evaluation [29,30]. Additionally, the precision of oral scanner machines and planning software were additional factors influencing the final outcomes [20,21]. Gimenez et al. indicated the accuracy might decrease with the increasing size of scanned sections and the improper operations [31,32]. It was worth emphasizing the presence of tolerance between the template, cylinder, drilling handle and drill, may cause inaccurate results too [20,33]. In a guided surgery, prefabricated cylinders with increasing diameter were embedded within the surgical template to accommodate different drilling handles, thus ensuring consecutive drilling bone sequence. Schneider et al. concluded the errors of surgical instruments could be reduced by using 3D-printed template with a reduced cylinder diameter, and the movement of drills could be further reduced by a shorter drill with a higher drill handle [22]. Thus, the design of surgical templates should be carefully considered to increase the accuracy of guided-surgeries. Some researchers also highlighted the positioning error of template on the support surface. During the cavity preparation and implant insertion, the template may move or rotate, inducing a positioning error, and the application of osteosynthesis screws may increase the accuracy of guided-surgeries [18]. The operator’s experience was also a considerable factor that may interfere the reality of the results. However, in a recent study, Cassette et al. have found that the experience of the operators has scarce influence on the guided surgeries [34].

An estimation of accuracy for full-guided and half-guided templates was meaningful as it provided a suggestion to clinicians when conducting guided-surgeries. Although we controlled some confounding factors that may interfere the results of the study, such as using the same tooth-supported template made in the same factory, the same implant system and the same operator, the argumentation intensity of the result was still not as good as a prospective study. Furthermore, the bone quality of patients was not determined, which may influence the angular deviation in half-guided surgeries using free-hand placement [35]. The different software used for full-guided and half-guided surgery in our study also may induce a bias. In addition to the limited sample size, short follow-up period, intrinsic errors of templates and various implant dimensions, further studies are urgently needed to confirm the findings of this study.

## 5. Conclusions

Within the limitations of this study, it could be concluded that there was no difference on the accuracy of guided-surgeries between anterior immediate and delayed implantations. Additionally, the full-guided template was more accurate than the half-guided template in anterior immediate implant surgery concerning the angular and depth deviation, while in delayed implant placement, full-guided surgical templates only performed better with statistical significance in depth deviations. What evidence there is tends to indicate that full-guided templates are more prudent for the anterior esthetic zone, but important fundamentals such as case selection, adequate safety distance and careful execution should not be forgotten forever.

## Figures and Tables

**Table 1:** Details of patients and treatments characteristics.

Group	No. Patients (Ratio)	Sex (Ratio)	Age		Implant Sites						
			Mean ± SD	Range	11/21	12/22	13/23	31/41	32/42	33/43	Total
Total patients	63 (-)	34 (54%)	40.35 ± 13.74	18–70	43	27	8	2	5	1	76
Full-guided template	30 (48%)	16 (53%)	40.87 ± 13.87	18–70	21	9	3	1	3	0	37
Half-guided template	33 (52%)	18 (55%)	39.88 ± 13.61	18–61	22	8	5	1	2	1	39
Immediate implantation	32 (51%)	19 (59%)	40.72 ± 13.75	18–63	25	8	1	0	5	1	40
Full-guided template	13 (41%)	9 (69%)	40.69 ± 13.16	18–63	10	4	0	0	3	0	17
Half-guided template	19 (59%)	10 (53%)	40.74 ± 14.14	18–61	15	4	1	0	2	1	23
Delayed implantation	31 (49%)	15 (48%)	39.97 ± 13.72	18–70	18	9	7	2	0	0	36
Full-guided template	17 (55%)	7 (41%)	41.00± 14.39	18–70	11	5	3	1	0	0	20
Half-guided template	14 (45%)	8 (57%)	38.71 ± 12.75	23–58	7	4	4	1	0	0	16

**Table 2:** The deviation between the planned and actual implant positions.

Group	Global Coronal Deviation			Global Apical Deviation			Depth Deviation			Angular Deviation		
	Mean ± SD (mm)	Range (mm)	*p*-Value	Mean ± SD (mm)	Range (mm)	*p*-Value	Mean ± SD (mm)	Range (mm)	*p*-Value	Mean ± SD (°)	Range (°)	*p*-Value
Immediate implantation	0.91 ± 0.63	0.10–3.10	0.057	1.23 ± 0.63	0.40–3.30	0.980	0.73 ± 0.66	0.10–3.10	0.063	2.20 ± 1.40	0.30–6.20	0.353
Delayed implantation	0.71 ± 0.41	0.10–1.80		1.23 ± 0.54	0.20–2.60		0.49 ± 0.41	0.00–1.80		2.52 ± 1.56	0.20–6.30	
Full-guided template	0.59 ± 0.28	0.10–1.30	0.010 *	0.99 ± 0.41	0.20–1.80	<0.001 ***	0.38 ± 0.26	0.00–1.10	<0.001 ***	1.91 ± 1.02	0.20–4.20	0.008 **
Half-guided template	1.04 ± 0.64	0.10–3.10		1.46 ± 0.64	0.50–3.30		0.84 ± 0.68	0.10–3.10		2.77 ± 1.72	0.40–6.30	
Immediate implantation												
Full-guided template	0.66 ± 0.26	0.10–1.00	0.005 **	0.96 ± 0.41	0.20–1.60	0.022 *	0.46 ± 0.24	0.00–0.80	0.013 **	1.69 ± 0.94	0.20–3.30	0.034 *
Half-guided template	1.10 ± 0.76	0.10–3.10		1.43 ± 0.70	0.50–3.30		0.93 ± 0.79	0.10–3.10		2.57 ± 1.57	0.40–6.30	
Delayed implantation												
Full-guided template	0.53 ± 0.29	0.20–1.30	0.509	1.10 ± 0.42	0.40–1.80	0.006 **	0.32 ± 0.26	0.10–1.10	0.008 **	2.09 ± 1.07	0.50–4.20	0.082
Half-guided template	0.94 ± 0.43	0.30–1.80		1.51 ± 0.55	0.60–2.20		0.71 ± 0.47	0.10–1.80		3.06 ± 1.92	0.50–5.59	

Note: * *p* < 0.05, ** *p* < 0.01, *** *p* < 0.001. Statistical analyzing units were implants, instead of patients.

**Table 3:** Coronal and apical deviations of full-guided and half-guided templates adjusting for depth deviation.

Variable	Coronal Deviation			Apical Deviation		
	Immediate Implantation	Delayed Implantation	Total Cases	Immediate Implantation	Delayed Implantation	Total Cases
Full-guided vs. half-guided	−0.012	−0.126	−0.052	−0.143	−0.345	−0.206
Depth deviation	0.917 ***	0.746 ***	0.873 ***	0.703 ***	0.408	0.600 ***
Intercept	0.250 ***	0.417 ***	0.306 ***	0.780 ***	1.224 ***	0.962 ***
N	40	36	76	40	36	76

Note: * *p* < 0.05, ** *p* < 0.01, *** *p* < 0.001. Statistical analyzing units were implants, instead of patients.
